# Maturation of coagulation factor IX during Xase formation as deduced using factor VIII‐derived peptides

**DOI:** 10.1002/2211-5463.12653

**Published:** 2019-07-02

**Authors:** Han Fang, Thomas Zögg, Hans Brandstetter

**Affiliations:** ^1^ Department of Biosciences University of Salzburg Austria; ^2^ VIB‐VUB Center for Structural Biology Brussels Belgium

**Keywords:** blood coagulation factors, conformational activation, factor VIII mimetic peptides, proteolytic activation, thrombophilic Padua mutant, Xase complex

## Abstract

Blood coagulation involves extrinsic and intrinsic pathways, which merge at the activation step of blood coagulation factor X to factor Xa. This step is catalysed by the extrinsic or intrinsic Xase, which consists of a complex of factor VIIa and its cofactor tissue factor or factor IXa (FIXa) and its cofactor coagulation factor VIIIa (FVIIIa). Upon complex formation with FVIIIa, FIXa is conformationally activated to the Xase complex. However, the mechanistic understanding of this molecular recognition is limited. Here, we examined FVIIIa‐FIXa binding in the context of FIXa's activation status. Given the complexity and the labile nature of FVIIIa, we decided to employ two FVIII‐derived peptides (558‐loop, a2 peptide) to model the cofactor binding of FIX(a) using biosensor chip technology. These two FVIII peptides are known to mediate the key interactions between FVIIIa and FIXa. We found both of these cofactor mimetics as well as full‐length FVIIIa bind more tightly to zymogenic FIX than to proteolytically activated FIXa. Consequently and surprisingly, we observed that the catalytically inactive FIX zymogen can outcompete the activated FIXa from the complex with FVIIIa, resulting in an inactive, zymogenic Xase complex. By contrast, the thrombophilic Padua mutant FIXa‐R170 in complex with the protein–substrate analogue BPTI bound tighter to FVIIIa than to the zymogen form FIX‐R170L, suggesting that the active Xase complex preferentially forms in the Padua variant. Together, these results provide a mechanistic basis for the thrombophilic nature of the FIX‐R170L mutant and suggest the existence of a newly discovered safety measure within the coagulation cascade.

**Abbreviations**
BPTI: basic pancreatic trypsin inhibitor
Factor IXa: activated factor IX
IB: inclusion body
PMSF: phenylmethylsulfonyl fluoride

Blood coagulation is a cascade‐like molecular system to stop blood loss upon vessel injury. The reaction cascades can be subdivided into an extrinsic and intrinsic pathway, which serve to initiate, maintain and eventually stop blood coagulation. Both pathways merge at the activation step of blood coagulation factor X to factor Xa which is catalysed by the extrinsic or intrinsic Xase, consisting of the complex of factor VIIa and its cofactor tissue factor or factor IXa and its cofactor VIIIa, respectively 1, 2, 3.

The homologous coagulation factors IX and X are trypsin‐like serine proteases, which obligatorily require the protein cofactors factor VIII and factor V for their clotting activity. Also the cofactors V and VIII share an homologous domain architecture, consisting of three paralogous A domains (A1, A2, A3) and two C domains (C1, C2) (Fig. 1). While the C domains confer binding to the membrane of activated platelets, the A domains mediate binding to the substrate (A1) and the protease (A2, A3). A so‐called B domain of variable length and post‐translational modification is interspersed between the A2 and A3 domains. The A2 domain of factor VIIIa and factor Va is particularly important as it conformationally activates the corresponding coagulation protease, that is factor IXa in complex with factor VIIIa and factor Xa in complex with factor Va. These two protein complexes activate the downstream substrates factor X and prothrombin and are accordingly termed Xase and prothrombinase 2, 3, 4, 5.

**Figure 1:**
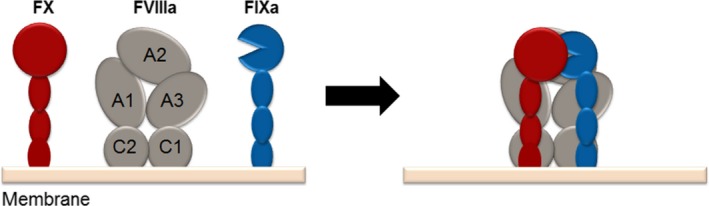
Factor VIII assists in orienting the FIXa‐FX interaction. Physiologically, FIXa acts on the activated platelets’ membrane surface, which expose negatively charged lipids during plug formation. FIXa has a very low amidolytic activity; only in the presence of its cofactor, factor VIIIa, the activity of FIXa is significantly increased. FVIIIa enhances both *k*_cat_ and *K*_M_ of the FIXa‐catalysed FX activation. In this instance, FVIIIa displays considerable affinity to both enzyme (FIXa) and substrate (FX) and pre‐orients both in a productive interaction.

All protein factors participating in the Xase and prothrombinase complexes require proteolytic activation to achieve enzymatic activity. Activation of factor VIII to VIIIIa, and correspondingly V to Va, is carried out by thrombin. Thrombin cleavages of factor VIII (and analogously in factor V) release the B domain by cleaving after Arg1689 and Arg740 (FV: Arg709, Arg1018 and Arg1545; sequence numbering of the mature proteins after cleavage of the signal peptides), and followed by the cleavage of the A1‐A2 linker after Arg372 in FVIII 6.

In the protease factors IX and X, proteolytic activation at Arg180 (FX: Arg194) and Arg145 releases an activation peptide, triggering a conformational disorder‐to‐order transition in the serine protease domain (Figs 2 and S1). The activation peptide is positioned between the light chain (consisting of the N‐terminal Gla, EGF1 and EGF2 domains) and the trypsin‐like serine protease domain. Light and heavy chains remain covalently linked via a disulfide bond. In both factor IX and factor X, the activation peptide harbours an intriguing high‐affinity binding site for benzamidine/arginine, exceeding the affinity towards the active site by an order of magnitude 7. By contrast, macromolecular factor IX/X inhibitors such as serpins or Kunitz inhibitors such as BPTI do not bind to the activation peptide but to the active site exclusively.

**Figure 2:**
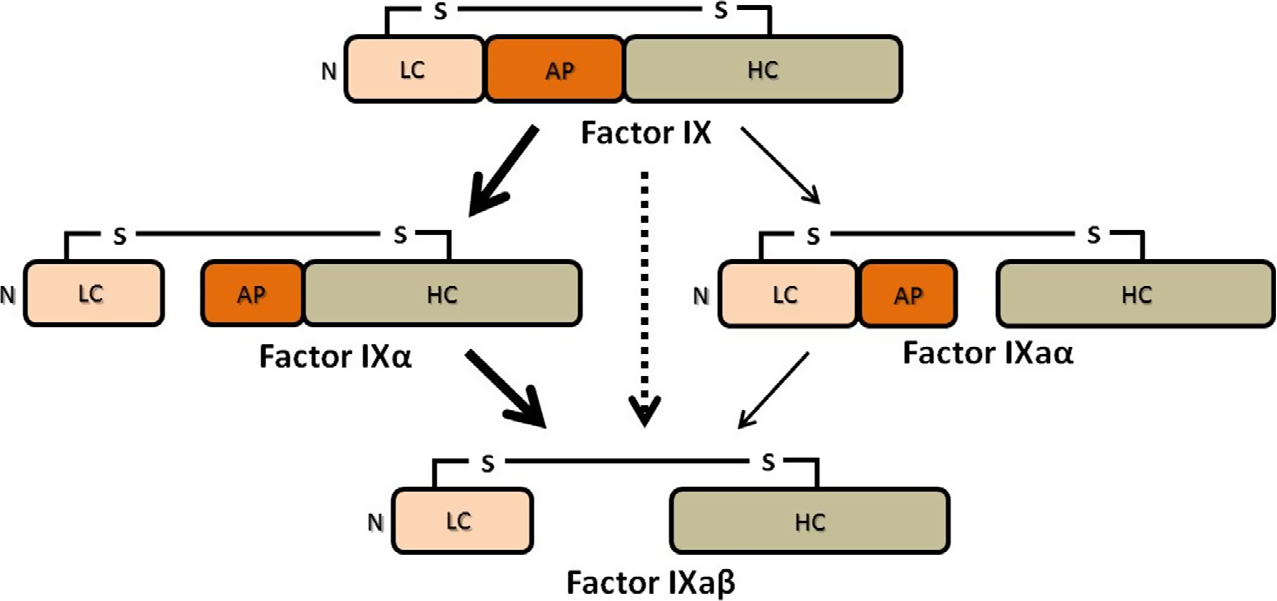
Release of the activation peptide (AP) during FIX activation. Dependent on the activator, the release of the AP can occur in two different orders, generating either enzymatically inactive factor IXα or active factor IXaα as single‐cleaved intermediate. Factor IX: zymogenic FIX. Factor IXα: nonenzymatic single‐cleaved FIX intermediate. Factor IXaα: enzymatic single‐cleaved FIX intermediate. Factor IXaβ: enzymatically active, double‐cleaved FIX, which corresponds to FIXa.

The proteolytic activation of factor IX (or X) to IXa is insufficient to achieve significant Xase activity. Instead, the cofactor VIIIa is critically needed to achieve physiologically relevant Xase activity. However, the mechanism of cofactor stimulation is incompletely understood. Functional and structural studies of this central haemostatic complex are hampered, among others, by the difficulty to obtain factor VIII(a) in sufficient amount and quality. To shed light on the cofactor triggered activation, we set out to study the interaction of factor IX with the cofactor VIIIa and VIIIa‐derived peptides mimicking important interactions.

## Materials and methods

### Expression of FIX constructs in *Escherichia coli* cells and purification

Factor IX protein variants were expressed in *Escherichia coli*, refolded, purified and activated as described elsewhere 8, 9, 10.

### Factor VIII‐derived peptides

The factor VIII‐derived peptides Ser558‐Gln565 (SVDQRGNQ) and Lys713‐Arg740 (KHTGDsYsYEDSsYEDISAYLLSKNNAIEPR; sY: sulfotyrosine) were purchased from JPT Peptide Technologies. Quality was controlled by HPLC and mass spectrometry and confirmed to be of better than 90% homogeneity.

### Activation of FVIII with thrombin

pro‐FVIII was generously provided by Baxter Biosciences (Vienna, Austria). Human thrombin was purchased from Sigma. For activation, 100 μg FVIII lyophilisate was dissolved in ddH2O and spin‐dialysed by repeated concentration in 30 kDa cut‐off Amicon concentrators to a final concentration of ~ 0.2 mg·mL^−1^. FVIII was activated by thrombin similar to protocols described elsewhere 11, 12. Briefly, thrombin was dissolved in 20 mm Hepes, pH 7.5, 100 mm NaCl and 5 mm CaCl_2_. 1 unit of thrombin was added to 1000 units of FVIII (100 μg) and incubated for 15 min at 37 °C without shaking. The protein mixture was then dialysed by in total four centrifugation steps against a 4 °C cold wash buffer consisting of 50 mm Hepes, pH 7.4, 300 mm NaCl and 2.5 mm CaCl_2_. The reaction was stopped after the second concentration step by adding 20 μL 50 mm PMSF to inhibit thrombin. The activation of FVIIIa was controlled by SDS/PAGE to observe the A1, A2 and A3‐C1‐C2 at ~55 kDa, 40 kDa and 70 kDa, respectively.

### Surface acoustic wave measurements via SAM5 device (SAW Instruments)

#### Chip calibration

Prior to the chip calibration at 22 °C, the instrument was flushed with a flow rate of 40 μL·min^−1^ for 5 to 10 min. Chip calibration was performed using the SAM5 program with gain of 180 and a phase difference of 0.363.

#### SAM5 ligand online immobilization

The SAM5 instrument with a dextran‐COOH‐coated gold chip (NanoTemper Technologies) was equilibrated using filtered and degassed PBS running buffer, and the chip was calibrated as described above. 1‐ethyl‐3‐(3‐dimethylaminopropyl) carbodiimide (EDC) and N‐hydroxysuccinimide (NHS) were dissolved in ddH_2_O to a final concentration of 400 mm and 100 mm, respectively. To activate the chip, EDC and NHS were mixed at equal volume, resulting in the final concentrations of 200 mm EDC and 50 mm NHS. The mixture was rapidly placed into position A1 of the tray holder. If carried out properly, the baseline will increase. Subsequently, the ligand (protein ligands at 250–500 nm concentration, peptidic ligands at 100 μm to 1 mm concentration) was placed into the tray holder at position A1. The binding of the ligand to the dextran matrix is based on electrostatic interactions. After activation, the matrix presents negative charges. Therefore, positively charged ligands are required, that is the pH has to be adjusted smaller than the ligand's pI, but larger than pH 3.5 (pI > pH > pH 3.5). The increase in the baseline resulting from successful ligand coating is larger than that resulting from EDC‐NHS activation. To cap the unspecific protein binding sites, 1 m ethanolamine, pH 8.5, was applied into the tray holder at position A3. The system was equilibrated against the running buffer.

#### SAM5 ligand and protein interaction measurements

Firstly, pumps were washed using filtered and degassed running buffer (20 mm HEPES pH 7.5, 150 mm NaCl, 5 mm CaCl_2_). The flow rate was set to 40 μL·min^−1^. After a stable baseline was reached, protein was injected by using the SAM5 sequence master program in an increasing concentration series (10 nm to 250 nm). The sensogram SAM5 data were exported to Origin and TraceDrawer, allowing to analyse and quantify the protein binding affinities, as described in the SAM5 user manual.

## Results

Recombinant factor IX was activated to FIXa using factor XIa (Figs S2, S3) and factor VIII to FVIIIa using thrombin (Fig. S4).

### Interaction of factor VIII with zymogenic and activated factor IX and IXa variants

**Figure 3:**
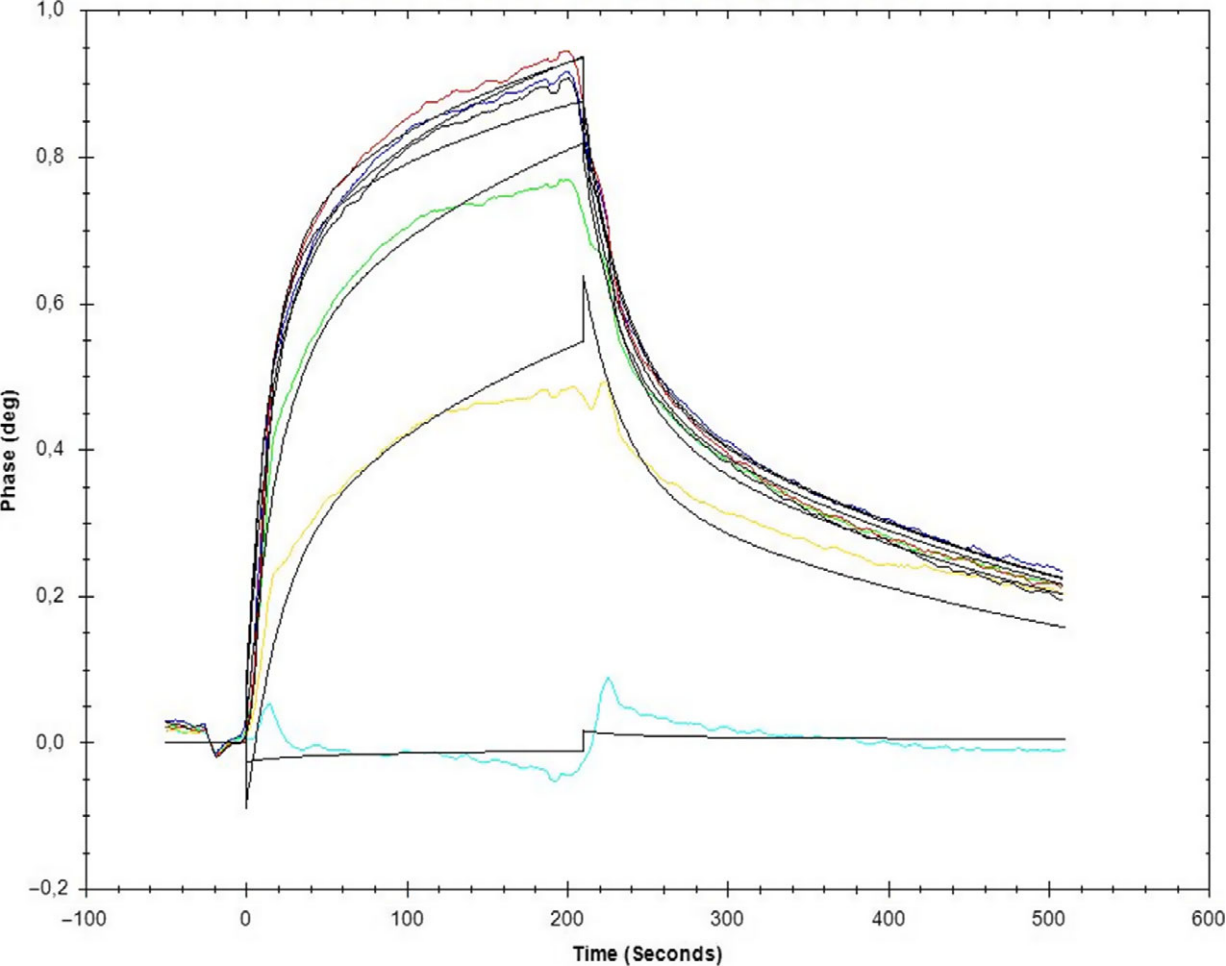
Sensogram of FIX binding to immobilized FVIII‐derived 558‐loop. FVIIIa‐derived peptide S558‐Q565 was immobilized on a carboxymethyl‐dextran chip, and zymogenic FIX wild‐type was applied in the mobile phase with six concentrations: 10 nm (light blue), 50 nm (yellow), 100 nm (green), 150 nm (dark blue), 200 nm (red) and 250 nm (black). The measurement was conducted at a flow rate of 40 μL·min^−1^ with 4‐min association with zymogenic FIX wild‐type, followed by 4‐min dissociation. For curve‐fitting, we used the one‐to‐one two‐state model, which is shown by the thin black lines.

The sequential order of factor IX and VIII activation and complex formation is not clearly established. Therefore, we asked whether not only the activated factor IXa but also the zymogenic factor IX would be able to bind coagulation factor VIIIa as well as its proform, factor VIII. We carried out the corresponding binding measurements with the cofactor being immobilized on a chip (Fig. 3). We found the zymogenic factor IX (FIX wt) to bind to the cofactor VIII with more than 10‐fold higher affinity than the activated factor IXa (FIXa wt). This was confirmed also when activated factor VIIIa was used as a binding partner, in the presence of both benzamidine and BPTI (Table**** 1).

**Table 1:** Dissociation constants of the interaction of FVIII or FVIIIa with various zymogenic FIX and activated FIXa constructs

FVIII analyte	Benzamidine *K*_d_ (nm)	FVIII analyte	Aprotinin (BPTI) *K*_d_ (nm)
*A*			
FIX wt	81.48 ± 86.77	FIX wt	65.77 ± 21.16
FIXa wt	948.00 ± 749.65	FIXa wt	107.62 ± 60.78
FIX‐R170L	40.43 ± 57.65	FIX‐R170L	46.66 ± 34.99
FIXa R170L	587.29 ± 937.70	FIXa R170L	73.10 ± 28.36
FIX 3M	50.64 ± 69.09	FIX 3M	56.00 ± 9.15
FIXa 3M	421.75 ± 392.66	FIXa 3M	192.25 ± 107.32
*B*			
FIX wt	83.62 ± 140.31	FIX wt	72.25 ± 60.21
FIXa wt	1304.13 ± 1381.53	FIXa wt	204.05 ± 169.08
FIX‐R170L	202.29 ± 404.48	FIX‐R170L	173.75 ± 281.43
FIXa R170L	249.89 ± 484.14	FIXa R170L	44.94 ± 32.26
FIX 3M	n.d.	FIX 3M	88.57 ± 76.30
FIXa 3M	817.75 ± 752.89	FIXa 3M	438.25 ± 250.87

The analytes were incubated with 1 mm benzamidine (left) or with 1 μm aprotinin/BPTI (right).

Section A describes the interaction of nonactivated factor VIII; section B describes that of activated factor VIIIa. Dissociation constants were determined using the SAM5 Blue device.

This finding was surprising to us and indicates that the tightest complex is the ‘pro‐Xase’ formed by the zymogenic FIX with either FVIII or FVIIIa. The preference of FVIII(a) binding to the inactive FIX may serve as another regulation level. Zymogenic factor IX and activated factor IXa not only differ by the presence of the activation peptide, but also conformationally, whereby the zymogen FIX is considerably more flexible and disordered as compared to activated FIXa 13, 14, 15. To further test the relevance of the factor IX(a) conformational rigidity for cofactor VIII(a) binding, we employed the triple factor IX mutant (Y94F‐K98T‐Y177T), which is known to be more active and conformationally more rigid than wt FIX 8. The binding of the pro‐cofactor VIII or activated cofactor VIIIa to the triple mutant factor IX (FIX 3M) in the presence of benzamidine or BPTI was qualitatively similar to the wt FIX(a), that is the zymogenic FIX‐3M bound with more than fourfold higher affinity than the activated FIXa‐3M, with FIX(a)‐3M having a slightly higher affinity than wt FIX(a) (Table 1).

Additionally, we investigated the thrombophilic Padua variant (R170L) which was suggested in the literature to have higher affinity to cofactor VIIIa 16. However, the mechanism of thrombophilicity and increased Xase activity is unknown. Overall, the Padua variant showed a similar binding pattern as the wt factor IX; however, we found one notable exception: in the wt FIX, the zymogen binds tighter to FVIII(a) than the activated FIXa independent of the ligand. By contrast, in the presence of BPTI we found the highest affinity for the Padua variant in the activated form FIXa(Padua) to the activated FVIIIa, that is the productive complex is preferred in the presence of a substrate analogue (Table 1), in stark contrast to other factor IX variants. The Padua variant thus prefers the complex formation with the proteolytically activated, that is enzymatically productive, components. This property may relate to the thrombophilic symptoms found in the Padua mutation.

To interpret these surprising findings, we further tried to reduce the interaction to known factor VIII interaction hotspots, that is the 558‐loop (Ser558‐Gln565) and the C‐terminal a2 peptide.

### Interaction of factor VIII‐derived 558‐loop with zymogenic and activated factor IX(a) variants

Albeit less pronounced, zymogenic and activated factor IX(a) variants exhibited analogous binding affinities towards the FVIII‐derived 558‐loop peptide as to protein cofactor VIII. The affinity of zymogenic wt FIX towards the 558‐loop was approximately threefold higher (*K*_d_ = 172 nm) than that of activated wt FIXa (*K*_d_ = 457 nm) (Table 2). A similar relation was observed for the triple mutant factor IX‐3M and the Padua variant (R170L): the zymogenic factor IX variants had an approximately twofold higher affinity towards the 558‐loop than the activated factor IXa variants (Table 2). These relations were also observed in the presence of benzamidine. Importantly, in the presence of the substrate analogue BPTI, the FIXa Padua variant had a higher binding affinity than the zymogenic FIX (Padua), mirroring the already described result for protein FVIIIa. The binding affinities of factor IX(a) to the 558‐loop thus parallel those observed for the binding towards the protein cofactor VIIIa.

**Table 2:** *K*_d_ values of FVIIIa A2 domain‐derived S558‐Q565 peptide (SVDQRGNQ, ‘558‐loop’) and different FIX(a) variants. The measurements were carried out without additive (left), or in the presence of 1 mm benzamidine (middle) and 1 μm aprotinin (BPTI) (right)

FVIIIa 558‐loop Analyte	No additive *K*_d_ (nm)	FVIIIa 558‐loop Analyte	Benzamidine *K*_d_ (nm)	FVIIIa 558‐loop Analyte	Aprotinin (BPTI) *K*_d_ (nm)
FIX wt	172.00 ± 37.63	FIX wt	501.60 ± 156.63	FIX wt	115.40 ± 34.62
FIXa wt	457.20 ± 121.14	FIXa wt	833.40 ± 270.93	FIXa wt	102.76 ± 25.00
FIX‐R170L	173.20 ± 57.15	FIX‐R170L	380.00 ± 115.53	FIX‐R170L	125.54 ± 103.99
FIXa R170L	374 ± 137.91	FIXa R170L	661.20 ± 183.98	FIXa R170L	70.46 ± 18.43
FIX 3M	111.04 ± 20.61	FIX 3M	99.08 ± 26.42	FIX 3M	67.00 ± 24.60
FIXa 3M	195.80 ± 39.68	FIXa 3M	274.20 ± 69.44	FIXa 3M	167.20 ± 57.58

### Interaction of factor VIII‐derived a2 peptide with zymogenic and activated factor IX(a) variants

**Figure 4:**
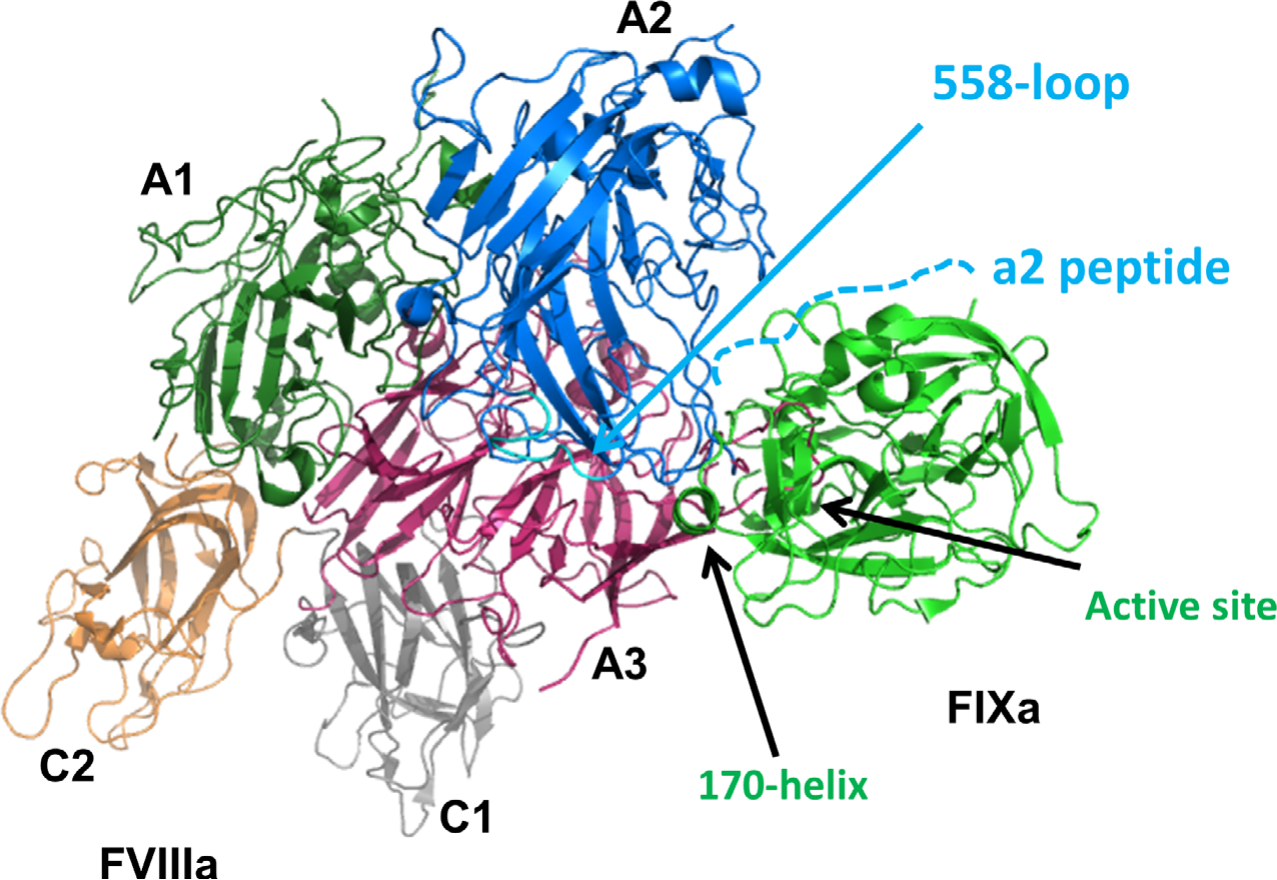
Interaction model of factor IXa with the cofactor VIIIa. The crystal structures of factor VIII (pdb entry 3CDZ) and factor IX (entry 5JB9) were superposed on the complex structure of the prothrombinase complex from *Pseudonaja textilis* (pdb entry 4BXS) to obtain a model of the human Xase complex. The structural model consistently explains biochemical data on the significance of the factor VIIIa‐derived 558‐loop and a2 peptide in the Xase complex 20, 21, 22.

We further investigated the binding to the a2 peptide, located at the C terminus of the A2 domain (Lys713‐Arg740), which is also known to contribute binding affinity to the Xase complex by interacting with the heparin‐binding site 17. We found that the a2 peptide had an approximately twofold higher affinity towards the zymogen factor IX than the activated factor IXa, independent from the presence of (substrate‐analogous) ligands or point mutations, that is FIX‐3M or FIX‐R170L (Padua) (Table 3). Different from the 558‐loop for the a2 peptide, we did not find a switch in the binding affinity of the zymogenic and activated Padua variants in the presence of BPTI (Tables 2 and 3). This suggests that the 558‐loop plays the dominant role in tuning the activation status of the Xase complex. This conclusion is consistent with the proposed binding sites of the 558‐loop and the a2 peptide. According to the *Pseudonaja textilis* prothrombinase complex, the 558‐loop binds to the 170‐helix of FIXa, in close proximity to the active site, whereas the a2 peptide binds to the heparin‐binding site of FIX, more distant to the active site 17 (Fig. 4).

**Table 3:** *K*_d_ values of the FVIIIa‐derived a2 peptide (KHTGDsYsYEDSsYEDISAYLLSKNNAIEPR) with different FIX(a) variants (‘Analyte’). The measurements were carried out without additive (left), or in the presence of 1 mm benzamidine (middle) or 1 μm BPTI (right)

FVIIIa – a2 analyte	No additive *K*_d_ (nm)	FVIIIa – a2	Benzamidine *K*_d_ (nm)	FVIIIa – a2	BPTI *K*_d_ (nm)
FIX wt	65.82 ± 23.48	FIX wt	59.04 ± 17.63	FIX wt	64.75 ± 99.83
FIXa wt	98.88 ± 11.61	FIXa wt	103.92 ± 21.80	FIXa wt	132.15 ± 62.22
FIX‐R170L	52.76 ± 9.75	FIX‐R170L	63.86 ± 11.05	FIX‐R170L	38.10 ± 5.32
FIXa R170L	102.94 ± 16.58	FIXa R170L	107.02 ± 31.83	FIXa R170L	114.83 ± 59.94
FIX 3M	16.03 ± 11.27	FIX 3M	35.65 ± 15.21	FIX 3M	52.45 ± 6.04
FIXa 3M	88.12 ± 37.94	FIXa 3M	118.18 ± 95.11	FIXa 3M	228.00 ± 181.57

## Discussion

The here described binding affinities of zymogenic and activated factor IX to factor VIII(a) and factor VIII‐derived peptides are partly surprising, but can be rationalized when considering the conformational transitions in factor IX.

Like all chymotrypsinogen‐like proteases, factor IX undergoes a conformational disorder–order transition upon proteolytic activation. Almost one third of the serine protease domain is conformationally flexible in the zymogenic state, including the activation pocket and the surrounding of the active site 18. The proteolytic activation of factor IX to factor IXa thus involves surface patches that are proposed to participate in cofactor binding (Fig. 4) 17. Therefore, it was to be expected that activation of factor IX to factor IXa should affect cofactor‐binding affinity, which was indeed the case, case **(**Tables**** 1 and 2). Additional conformational transitions are induced in activated factor IXa by binding to peptidic substrates, which are known to further mature the active site region 19. Consequently, substrate binding should also influence the binding affinity towards factor VIII(a). We mimicked the substrate‐bound factor IXa by using the canonical (i.e. substrate‐like) protein inhibitor BPTI and observed a change in binding affinity towards the activated cofactor VIIIa or the VIIIa‐derived peptide Ser558‐Gln565. In all factor IXa variants, the presence of BPTI significantly improved the affinity towards FVIIIa.

**Figure 5:**
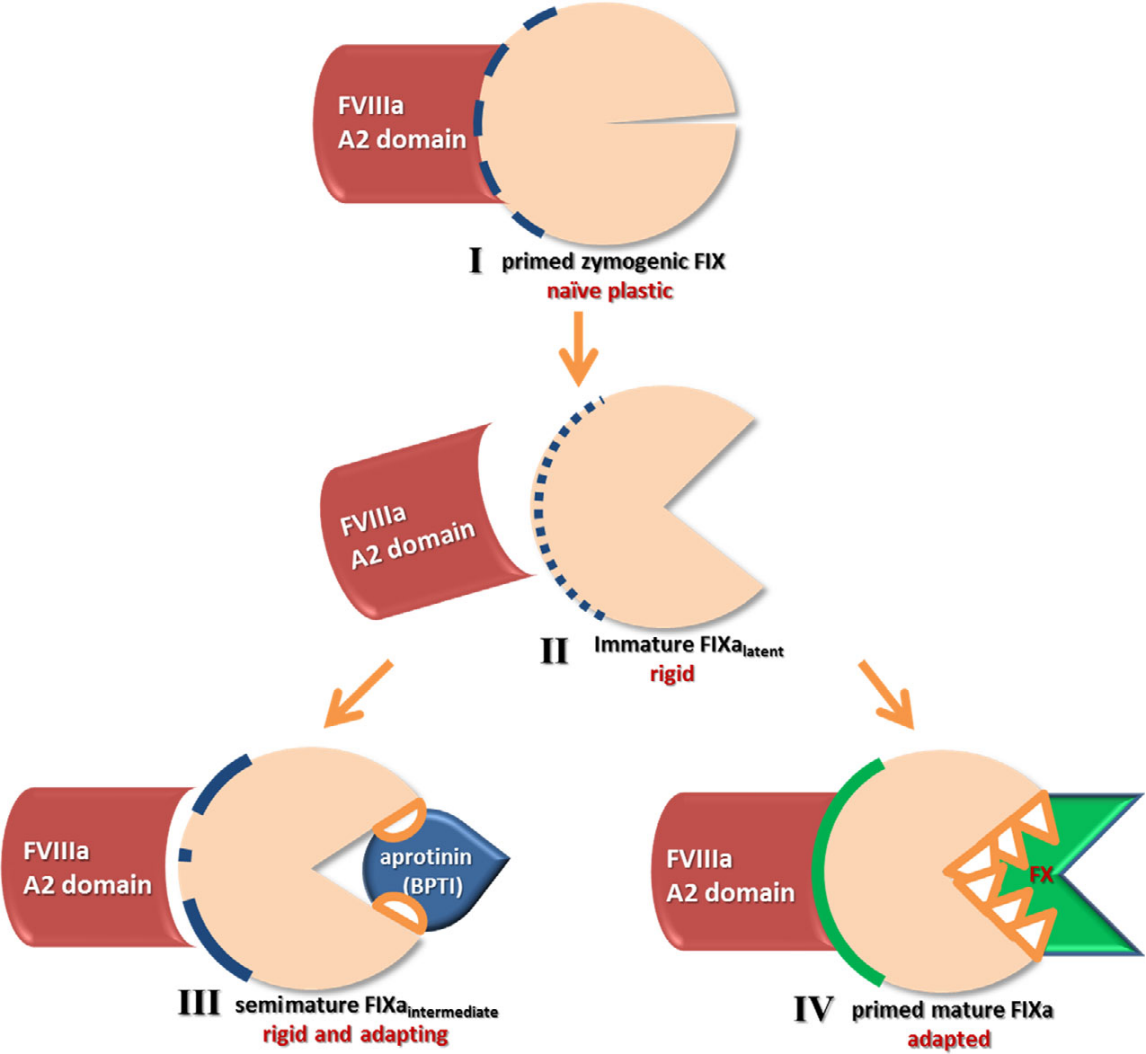
Illustration of FIXa maturation and interaction with FVIIIa A2 domain and substrates. I. Primed zymogenic FIX is plastic; it can flexibly adapt (dashed blue interface) to interact with the FVIIIa A2 domain. II. Immature FIXa_latent_ is rigid; the dotted interface (blue) indicates a poor compatibility with the cofactor FVIIIa‐derived A2 domain. III. The protein–substrate analogue BPTI converts FIXa_latent_ into FIXa_intermediate_ and increases the cofactor FVIIIa binding affinity, as indicated by the mostly continuous interface (blue). IV. The natural substrate of FIXa, FX, will be able to prime and shape the factor IXa interface for best cofactor binding, as indicated by the solid green line.

In summary, we propose a four‐state model to explain the observed results (Fig. 5): zymogenic FIX (I) is enzymatically inactive, conformationally flexible and plastic. Upon proteolytic activation, FIX is converted to FIXa_latent_ [immature FIXa (II)], which has its active site preformed, and is conformationally rigidified, thereby losing its plasticity. Importantly, however, the conformation of FIXa_latent_ is partly incompatible with cofactor binding, and it takes energy to adapt to the cofactor‐binding conformation, thus reducing the affinity of binding. By contrast, zymogenic FIX (I) can more easily adapt to the conformation consistent with cofactor binding. The conformation of FIXa(II) is modulated in the complex with the protein–substrate analogue BPTI (III). Different from benzamidine, BPTI has an extensive interaction interface with FIXa and is thereby able to convert FIXa_latent_ (II) into FIXa_intermediate_ (III), resulting in approximately equal affinities of FIXa_intermediate_‐FVIIIa (III) and FIX‐FVIIIa (I). This assimilation in affinity is seen in the complex of FIX(a) with the 558‐loop (Ser558‐Gln565) and most pronounced in the complex with FVIII and FVIIIa, but not in the a2 peptide (Lys713 – Arg740). The latter observation is consistent with the proposed binding site of a2 to FIXa near the heparin‐binding site, away from the active site 17.

Remarkably, the clinically relevant thrombophilic mutant R170L is able to further improve the binding affinity of the activated FIXa towards FVIIIa in the ternary complex with the protein–substrate analogue BPTI, not benzamidine or the apo form. An analogous effect was observed for the FVIIIa‐derived 558‐loop, but not for the C‐terminal a2 peptide. This latter effect is consistent with the R170L mutation being positioned directly at the binding interface to the 558‐loop (Ser558‐Gln565), see Fig. 4, whereas the binding epitope of the a2 peptide (Lys713‐Arg740) is more than 30 Å away from the mutation site. This analysis rationalizes the molecular basis of the thrombophilic effect of the R170L mutation which is not caused by an increase in the binary FIXa‐FVIIIa binding affinity but by an increase in the binding affinity of the ternary FVIIIa‐FIXa substrate complex. Finally, we propose that the correct physiological substrate FX would be able to induce an even more compatible conformation in FIXa (IV).

## Conclusions

The conformational changes accompanying the proteolytic activation of factor IX to IXa affect not only its enzymatic activity but also its binding properties towards the cofactor VIIIa. Unexpectedly, the affinity of the complex of zymogen FIX towards the cofactor VIII(a) is higher than that of the activated FIXa to FVIIIa Xase complex. Consequently, the zymogenic ‘pro‐Xase’ outcompetes the catalytically active Xase, keeping blood coagulation under tight control. In the ternary complex with the additional presence of a substrate analogue, the Xase complex gets tightened, enabling an efficient substrate turnover. Intriguingly, the Padua variant of active factor IXa had a particularly strong affinity in the ternary complex, explaining its thrombophilic property.

## Conflict of interest

The authors declare no conflict of interest.

## Author contributions

HF conducted most experiments and wrote the manuscript. TZ supervised the work. HB supervised the work and wrote the manuscript.

## Supporting information


**Fig. S1.** Domain organization of the homologous factor IX and X. (A) Benzamidine binding sites in zymogenic factor IX / X. (B) Benzamidine binding sites in the activated factor IX a/ Xa.
**Fig. S2.** SDS/PAGE of the SEC fractions of zymogenic FIX wt. The sample containing FIX wt after Q sepharose purification is referred to as load. The most pure fractions were pooled and stored at ‐20 °C for further experiments.
**Fig. S3.** Zymogenic FIX wt activation by hFXIa. The *E. coli* expressed recombinant FIX wt was activated by hFXIa. The 4 lanes on the left side show samples loaded with dithiothreitol. The remaining 4 samples at the right side were without DTT. The samples without DTT showed an extra thin band on the top, which corresponds to dimeric hFXIa. Wild‐type and mutant zymogenic FIX were activated by human blood coagulation FXIa (hFXIa), cleaving at Arg145‐Ala146 and Arg180‐Val181. After the first cleavage (at the Arg145‐Ala146; after 1 h) an activation intermediate FIXα was observed, which migrated at an increased apparent size. Activation was completed after 16 hours, corresponding to resembling cleavages at position Arg145‐Ala146 and Arg180‐Val181, with the activation peptide being released.
**Fig. S4** FVIII activation via human thrombin. FVIII was incubated with human thrombin for 30 min at 37 °C without shaking to generate the activated form. The samples were loaded without (marked in blue) and with (marked in red) DTT. The B domain deleted pro‐FVIII with an approximate molecular mass of 280 kDa was activated by human thrombin and converted into three major bands: A1 domain approx. 50 kDa, A2 domain approx. 40 kDa, and A3‐C1‐C2 domain approx. 80 kDa.

## References

[1] Kolkman JA , Lenting PJ and Mertens K (1999) Regions 301‐303 and 333‐339 in the catalytic domain of blood coagulation factor IX are factor VIII‐interactive sites involved in stimulation of enzyme activity. Biochem J 339 (Pt 2), 217–221. 10191249 PMC1220147

[2] Lenting PJ , van Mourik JA and Mertens K (1998) The life cycle of coagulation factor VIII in view of its structure and function. Blood 92, 3983–3996. 9834200

[3] Ngo JC , Huang M , Roth DA , Furie BC and Furie B (2008) Crystal structure of human factor VIII: implications for the formation of the factor IXa‐factor VIIIa complex. Structure 16, 597–606. 18400180 10.1016/j.str.2008.03.001

[4] Fay PJ and Koshibu K (1998) The A2 subunit of factor VIIIa modulates the active site of factor IXa. J Biol Chem 273, 19049–19054. 9668086 10.1074/jbc.273.30.19049

[5] Hakeos WH , Miao H , Sirachainan N , Kemball‐Cook G , Saenko EL , Kaufman RJ and Pipe SW (2002) Hemophilia A mutations within the factor VIII A2‐A3 subunit interface destabilize factor VIIIa and cause one‐stage/two‐stage activity discrepancy. Thromb Haemost 88, 781–787. 12428094

[6] Camire RM and Bos MH (2009) The molecular basis of factor V and VIII procofactor activation. J Thromb Haemost 7, 1951–1961. 19765210 10.1111/j.1538-7836.2009.03622.x PMC2993324

[7] Griessner A , Zögg T and Brandstetter H (2013) The activation peptide of coagulation factor IX and X serves as a high affinity receptor to cationic ligands. Thromb Haemos 110, 620–622.

[8] Zogg T and Brandstetter H (2009) Structural basis of the cofactor‐ and substrate‐assisted activation of human coagulation factor IXa. Structure 17, 1669–1678. 20004170 10.1016/j.str.2009.10.011

[9] Fang H (2018) Structural and biochemical characterization of coagulation factors VIIIa and IXa In Biosciences. University of Salzburg, Salzburg.

[10] Kristensen LH , Olsen OH , Blouse GE and Brandstetter H (2016) Releasing the brakes in coagulation Factor IXa by co‐operative maturation of the substrate‐binding site. Biochem J 473, 2395–2411. 27208168 10.1042/BCJ20160336

[11] Lollar P , Fay PJ and Fass DN (1993) Factor VIII and factor VIIIa. Methods Enzymol 222, 128–143. 8412790 10.1016/0076-6879(93)22010-d

[12] Lollar P , Knutson GJ and Fass DN (1985) Activation of porcine factor VIII: C by thrombin and factor Xa. Biochemistry 24, 8056–8064. 3937553 10.1021/bi00348a033

[13] Sichler K , Banner DW , D'Arcy A , Hopfner KP , Huber R , Bode W , Kresse GB , Kopetzki E and Brandstetter H (2002) Crystal structures of uninhibited factor VIIa link its cofactor and substrate‐assisted activation to specific interactions. J Mol Biol 322, 591–603. 12225752 10.1016/s0022-2836(02)00747-7

[14] Sichler K , Kopetzki E , Huber R , Bode W , Hopfner KP and Brandstetter H (2003) Physiological fIXa activation involves a cooperative conformational rearrangement of the 99‐loop. J Biol Chem 278, 4121–4126. 12444082 10.1074/jbc.M210722200

[15] Walter J , Steigemann W , Singh TP , Bartunik H , Bode W and Huber R (1982) On the disordered activation domain in trypsinogen: chemical labelling and low‐temperature crystallography. Acta Crystal Sec B Struct Crystal Crystal Chem 38, 1462–1472.

[16] Simioni P , Tormene D , Tognin G , Gavasso S , Bulato C , Iacobelli NP , Finn JD , Spiezia L , Radu C and Arruda VR (2009) X‐linked thrombophilia with a mutant factor IX (factor IX Padua). N Engl J Med 361, 1671–1675. 19846852 10.1056/NEJMoa0904377

[17] Lechtenberg BC , Murray‐Rust TA , Johnson DJ , Adams TE , Krishnaswamy S , Camire RM and Huntington JA (2013) Crystal structure of the prothrombinase complex from the venom of Pseudonaja textilis. Blood 122, 2777–2783. 23869089 10.1182/blood-2013-06-511733 PMC3798993

[18] Bode W , Schwager P and Huber R (1978) The transition of bovine trypsinogen to a trypsin‐like state upon strong ligand binding. The refined crystal structures of the bovine trypsinogen‐pancreatic trypsin inhibitor complex and of its ternary complex with Ile‐Val at 1.9 A resolution. J Mol Biol 118, 99–112. 625059 10.1016/0022-2836(78)90246-2

[19] Lubin IM , Healey JF , Barrow RT , Scandella D and Lollar P (1997) Analysis of the human factor VIII A2 inhibitor epitope by alanine scanning mutagenesis. J Biol Chem 272, 30191–30195. 9374501 10.1074/jbc.272.48.30191

[20] Jagannathan I , Ichikawa HT , Kruger T and Fay PJ (2009) Identification of residues in the 558‐loop of factor VIIIa A2 subunit that interact with factor IXa. J Biol Chem 284, 32248–32255. 19801661 10.1074/jbc.M109.050781 PMC2781637

[21] Fay PJ (2006) Factor VIII structure and function. Int J Hematol 83, 103–108. 16513527 10.1532/IJH97.05113

[22] Jenkins PV , Dill JL , Zhou Q and Fay PJ (2004) Contribution of factor VIIIa A2 and A3‐C1‐C2 subunits to the affinity for factor IXa in factor Xase. Biochemistry 43, 5094–5101. 15109268 10.1021/bi036289p

