# Supplementary material for: Maturation of coagulation factor IX during Xase formation as deduced using factor VIII‐derived peptides
Source: FEBS Open Bio. 2019 Jul 2;9(8):1370–8. doi: 10.1002/2211-5463.12653 (PMC6668378; doi:10.1002/2211-5463.12653)
Supplement: Supplementary file 1 — Fig. S1. Domain organization of the homologous factor IX and X. (A) Benzamidine binding sites in zymogenic factor IX / X. (B) Benzamidine binding sites in the activated factor IX a/ Xa. Fig. S2. SDS/PAGE of the SEC fractions of zymogenic FIX wt. The sample containing FIX wt after Q sepharose purification is referred to as load. The most pure fractions were pooled and stored at ‐20 °C for further experiments. Fig. S3. Zymogenic FIX wt activation by hFXIa. The E. coli expressed recombinant FIX wt was activated by hFXIa. The 4 lanes on the left side show samples loaded with dithiothreitol. The remaining 4 samples at the right side were without DTT. The samples without DTT showed an extra thin band on the top, which corresponds to dimeric hFXIa. Wild‐type and mutant zymogenic FIX were activated by human blood coagulation FXIa (hFXIa), cleaving at Arg145‐Ala146 and Arg180‐Val181. After the first cleavage (at the Arg145‐Ala146; after 1 h) an activation intermediate FIXα was observed, which migrated at an increased apparent size. Activation was completed after 16 hours, corresponding to resembling cleavages at position Arg145‐Ala146 and Arg180‐Val181, with the activation peptide being released. Fig. S4 FVIII activation via human thrombin. FVIII was incubated with human thrombin for 30 min at 37 °C without shaking to generate the activated form. The samples were loaded without (marked in blue) and with (marked in red) DTT. The B domain deleted pro‐FVIII with an approximate molecular mass of 280 kDa was activated by human thrombin and converted into three major bands: A1 domain approx. 50 kDa, A2 domain approx. 40 kDa, and A3‐C1‐C2 domain approx. 80 kDa. [file FEB4-9-1370-s001.pdf]

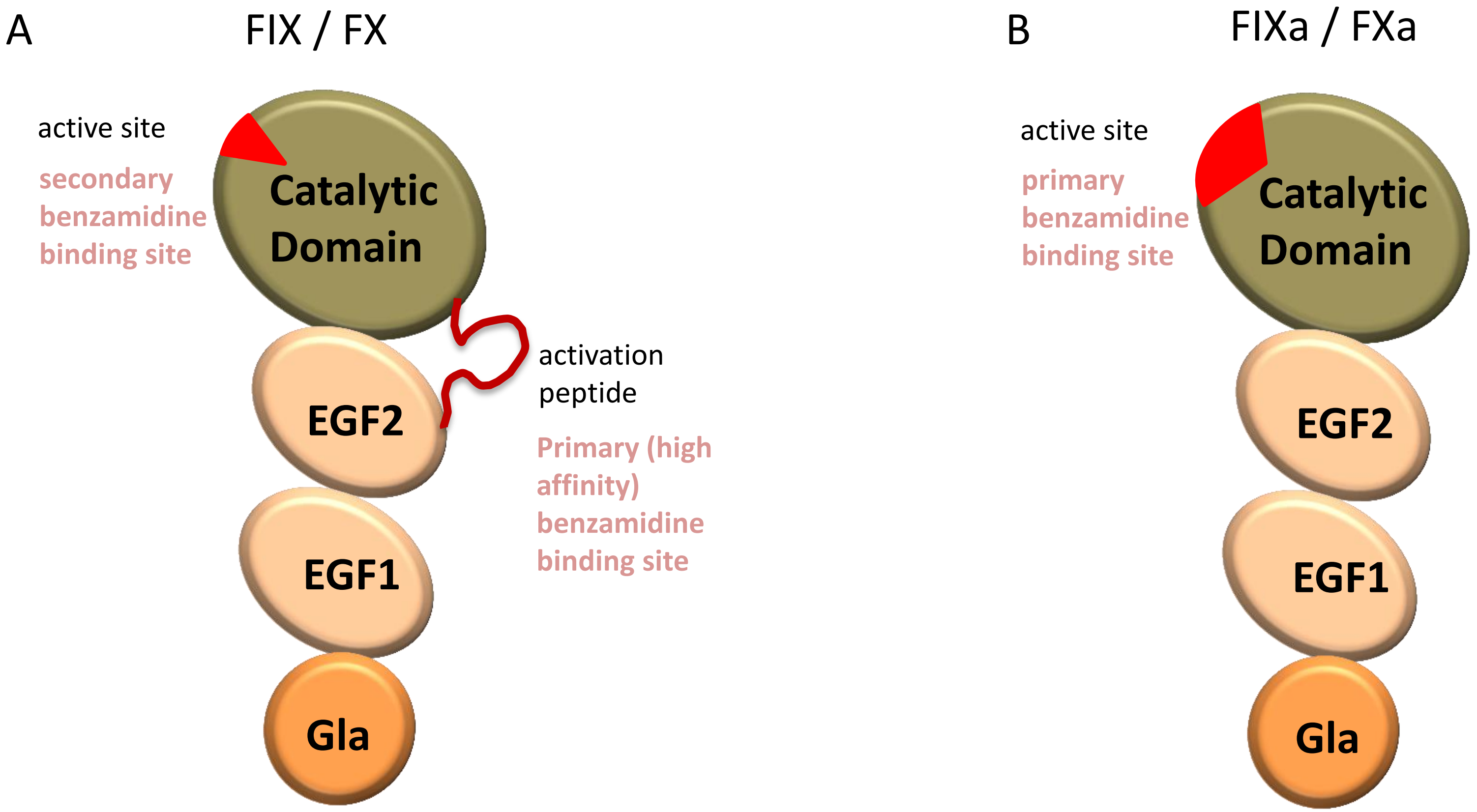

Supplementary Figure 1: Domain organization of the homologous factor IX and X

A) Benzamidine binding sites in zymogenic factor IX / X

B) Benzamidine binding sites in the activated factor IX a/ Xa

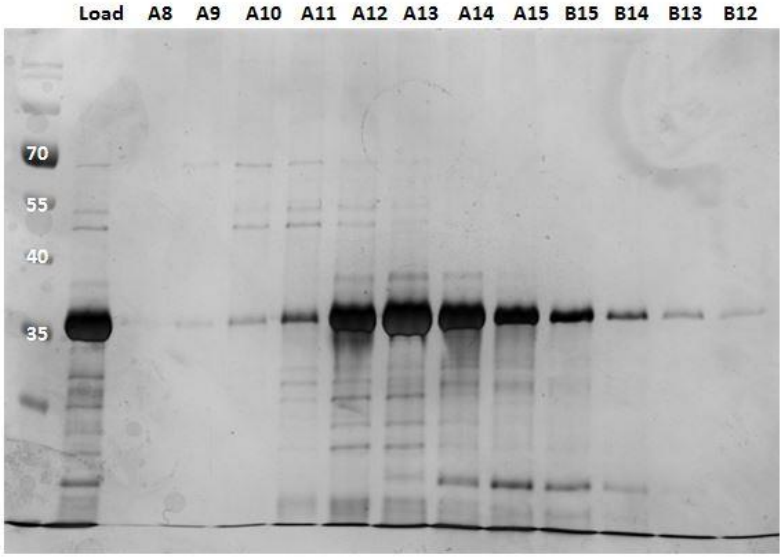

Supplemental figure 2: SDS-PAGE of the SEC fractions of zymogenic FIX wt.
The sample containing FIX wt after Q sepharose purification is referred to as load.. The most pure fractions were pooled and stored at -20 °C for further experiments.

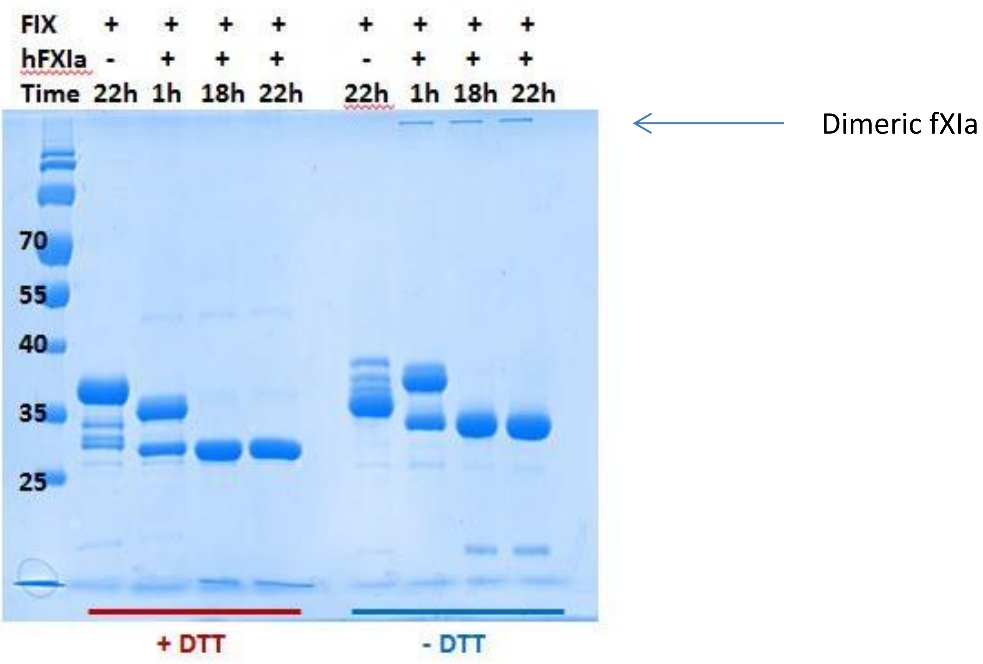

Supplemental figure 3: Zymogenic FIX wt activation by hFXIa.
The *E.coli* expressed recombinant FIX wt was activated by hFXIa. The 4 lanes on the left side show samples loaded with dithiothreitol. The remaining 4 samples at the right side were without DTT. The samples without DTT showed an extra thin band on the top, which corresponds to dimeric hFXIa. Wild type and mutant zymogenic FIX were activated by human blood coagulation FXIa (hFXIa), cleaving at Arg145-Ala146 and Arg180-Val181. After the first cleavage (at the Arg145-Ala146; after 1 h) an activation intermediate FIXα was observed, which migrated at an increased apparent size. Activation was completed after 16 hours, corresponding to resembling cleavages at position Arg145-Ala146 and Arg180-Val181, with the activation peptide being released.

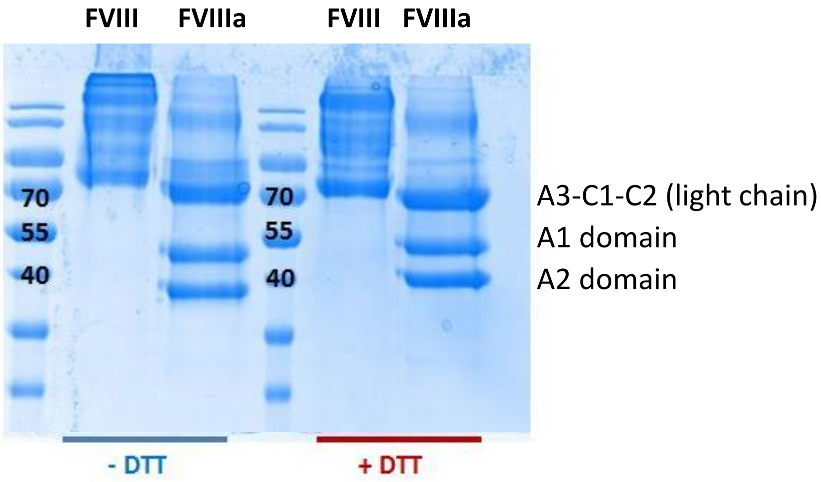

Supplemental figure 4: FVIII activation via human thrombin.
FVIII was incubated with human thrombin for 30 min at 37 °C without shaking to generate the activated form. The samples were loaded without (marked in blue) and with (marked in red) DTT. The B domain deleted pro-FVIII with an approximate molecular mass of 280 kDa was activated by human thrombin and converted into three major bands: A1 domain approx. 50 kDa, A2 domain approx. 40 kDa, and A3-C1-C2 domain approx. 80 kDa.
